# Massive circulating metastatic cells: A case of carcinocythemia

**DOI:** 10.1002/ccr3.8471

**Published:** 2024-02-07

**Authors:** Carine Farkh, Jérome Debus, Valérie Andrieu, Juliette Gay

**Affiliations:** ^1^ Laboratoire Hématologie, AP‐HP Hôpital Bichat‐Claude Bernard Paris France

**Keywords:** breast neoplasms, carcinocythemia, circulating tumor cells, hematologic malignancy, lymphocytic chronic B‐cell

## Abstract

Importance of careful differential diagnosis to make the distinction between carcinocythemia and acute leukemia or lymphoma.

## Graphical abstract

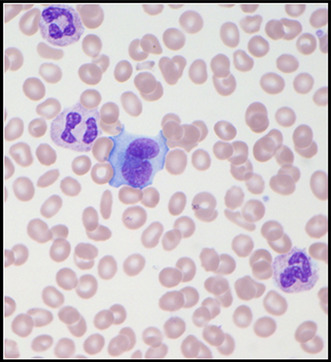

We report the case of an 80‐year‐old woman who presented to the emergency department with worsening chronic dyspnea and cervical adenopathy. Her medical history included chronic lymphocytic leukemia (CLL) diagnosed in 2012 with a Matutes score of 5, TP53 and NOTCH1 mutations and mutated IGHV genes. She was treated with Venetoclax since October 2020 after several treatment failures with ibrutinib, rituximab, and idelalisib due to bad tolerance. She had also a medical history of breast cancer, treated surgically in association with radiotherapy and hormone therapy, in remission for 6 years.

The whole blood count (white blood cells, 13.5 G/L; hemoglobin, 13 g/dL; platelets, 143 G/L) showed unusual interferences on the WDF scattergram of the XN‐series analyzer (Sysmex), with a high florescence cell population (Figure 1A) without cytopenia.

**FIGURE 1:**
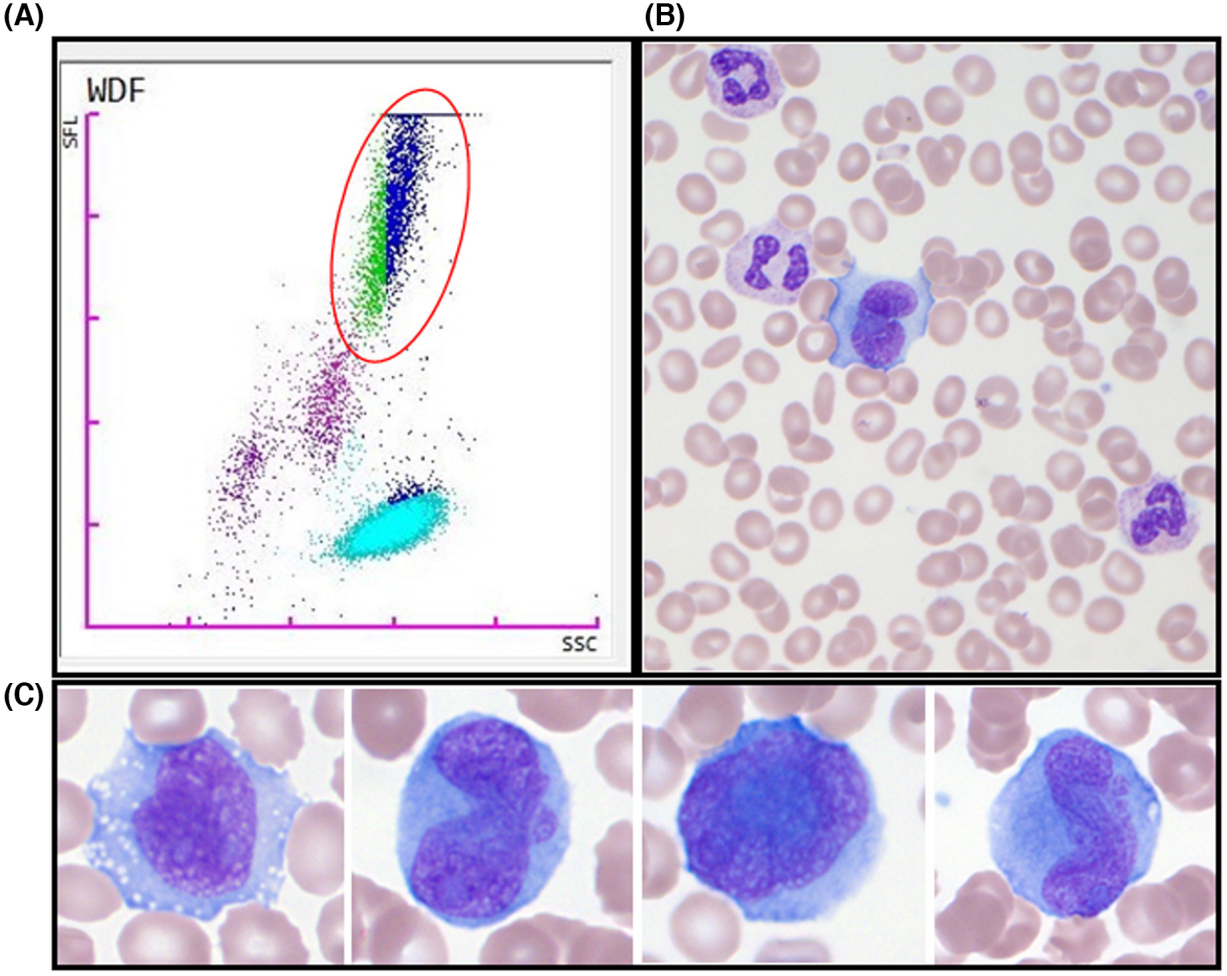
WDF scattergram XN‐series analyzer (Sysmex) and blood smear (May‐Griinwald‐Giemsa × 1000). Presence of circulating cancer cells.

The blood smear after May‐Grünwald Giemsa staining (original magnification ×100 and ×1000), showed 30% of discohesive, large, atypical cells with abundant basophilic vacuolated cytoplasm, irregular pseudolobed nuclei, with weak chromatin condensation and blue prominent nucleoli (Figure 1B,C).

The flow cytometry analysis showed the presence of 11% of kappa monotypic B cells with a phenotypic profile consistent with the known CLL and no evidence of a circulating phase of large cell lymphoma or blasts.

Altogether, these results ruled out hematological malignancies and showed the presence of atypical non‐hematopoietic cells in a known case of CLL.

This case points toward a breast cancer recurrence and lymphangitis carcinomatosis, the atypical non‐hematopoietic cells were circulating cancer cells (carcinocytemia). Unfortunately, the patient developed disseminated intravascular coagulation and died shortly thereafter, before additional exploration.

Only few cases of carcinocythemia have been described in the literature.^1^, ^2^, ^3^, ^4^ The most frequent primary neoplasm associated with carcinocythemia is breast carcinoma. The proportion of circulating metastatic cells vary from single cell to 80% of circulating nucleated cells mimicking myeloid or lymphoid neoplasm.^1^, ^3^ It is associated with poor prognosis and survival with 85% of mortality in the literature series and 34.5% reported cases of carcinocythemia were associated with intravascular coagulation or thrombotic events, leading to death similar to our case.^1^

This rare case of massive circulating metastatic cells highlights the importance of careful differential diagnosis to make the distinction between carcinocythemia and acute leukemia or lymphoma.

## AUTHOR CONTRIBUTIONS

**Carine Farkh:** Conceptualization; data curation; formal analysis; investigation; methodology; writing – original draft; writing – review and editing. **Jérome Debus:** Conceptualization; data curation; formal analysis; investigation; writing – review and editing. **Valérie Andrieu:** Conceptualization; data curation; investigation; writing – review and editing. **Juliette GAY:** Conceptualization; data curation; formal analysis; investigation; methodology; supervision; writing – original draft; writing – review and editing.

## FUNDING INFORMATION

None.

## CONFLICT OF INTEREST STATEMENT

The authors declare no conflicts of interest.

## CONSENT

Written informed consent was obtained from the patient to publish this report in accordance with the journal's patient consent policy.

## Data Availability

The author confirm that the data supporting the findings of this study are available within the article.
